# Supplementary material for: LPA1 Receptor Promotes Progesterone Receptor Phosphorylation through PKCα in Human Glioblastoma Cells
Source: Cells. 2021 Apr 4;10(4):807. doi: 10.3390/cells10040807 (PMC8066126; doi:10.3390/cells10040807)
Supplement: Supplementary file 1 [file cells-10-00807-s001.pdf]

Table S1. Characteristics of the antibodies

Information on the antibody					
**Name of the antibody**	**Manufacturer, catalogue#, batch #**	**Protein target**	**Antigen sequence**	**Species raised, monoclonal/polyclonal**	**Dilution used**
PKCα Antibody (H-7)	Santa Cruz Biotechnology Catalog# sc-8393 Batch# 12909	PKCα of mouse, rat and human origin	amino acids 645-672 at the C-terminus of PKC α of human origin	Mouse monoclonal IgG_1	1 μg/mL or 2 μg/mL
Anti-rabbit IgG (H+L) Alexa Fluor 488	Invitrogen by Thermo Fisher Scientific Catalog# A11034 Batch# 19107995C	Affinity purified goat anti-rabbit IgG whole antibodies have been highly cross-adsorbed against bovine IgG, goat IgG, mouse IgG, rat IgG, and human IgG	Rabbit IgG	Goat polyclonal IgG	0.5 μg/mL
Progesterone Receptor (H-190)	Santa Cruz Biotechnology Catalog# sc-7208 Batch# A1711	Progesterone receptor isoforms A and B of human origin	Polyclonal antibody raised against amino acids 375-564 of PR of human origin	Rabbit polyclonal	2 μg/mL
pS400 Progesterone Receptor	Abcam Catalog# ab60954 Batch# GR85615-1	Human progesterone receptor phosphorylated at serine 400	Synthetic phosphor peptide derived from human Progesterone Receptor around the phosphorylation site of serine 400 ((A-R-S^P-P-R).	Rabbit polyclonal	1 μg/mL
